# Supplementary material for: TADfit is a multivariate linear regression model for profiling hierarchical chromatin domains on replicate Hi-C data
Source: Commun Biol. 2022 Jun 20;5:608. doi: 10.1038/s42003-022-03546-y (PMC9209495; doi:10.1038/s42003-022-03546-y)
Supplement: Supplementary file 2 — Supplementary Information [file 42003_2022_3546_MOESM2_ESM.pdf]

Supplementary Information for

TADfit is a multivariate linear regression model for profiling hierarchical chromatin domains on replicate Hi-C data

Erhu Liu[1], Hongqiang Lyu[1,3*], Qinke Peng[1], Yuan Liu[1], Tian Wang[2] & Jiuqiang Han[3,1]

[1]School of Automation Science and Engineering, Faculty of Electronic and Information Engineering, Xi'an Jiaotong University, Shaanxi 710049, China

[2]Institute of Artificial Intelligence, Beihang University, Beijing 100191, China

[3]Guangdong Artificial Intelligence and Digital Economy Laboratroy, Guangdong 510335, China

[*]Correspondence and requests for materials should be addressed to H.L. (email: hongqianglv@mail.xjtu.edu.cn)

Contents:

I. Supplementary Notes

II. Supplementary Figures

III. Supplementary Tables

IV. Supplementary References

I. Supplementary Notes

Supplementary Note 1 Algorithm of TADfit model and FTRL solver

The proposed multivariate linear regression model TADfit is used for profiling hierarchical TADs on replicate Hi-C contact matrices. The pseudo code for TADfit model and FTRL [1,2] solver is as follows:

TADfit ($A, winsize, maxsize, minsize, repeat, iteration, \alpha, \beta, l$)

Input: A is a list of k contact matrix replicates with a dimension of $m \times m$, $winsize$ is a window size engaged in TopDom, $maxsize$ and $minsize$ are two thresholds giving the maximum and minimum size of TADs that can be identified, respectively, $repeat$ and $iteration$ separately denote the number of repetitions of solving the regression coefficients and the number of iterations in each solving by FTRL while moving toward a minimum of loss function, α, β and l are hyperparameters involved in FTRL solver, the first two are for per-coordinate learning rate, and the last one is for the strength of L_1 regularization.

Output: C_s is a set of significant hierarchical TADs,
B_s is the regression coefficients of the hierarchical TADs in C_s.

$\mathbf{A}_p = (A_1 \bullet A_1 \bullet \ldots \bullet A_1)^{1/k}$ //Construction of pseudo contact matrix
$\mathbf{T}$ = TopDom($\mathbf{A}_p$, $winsize$) //Calling TAD boundaries by TopDom
C=null //Initialization of candidate hierarchical TAD
for $i = 1$ to length($\mathbf{T}$) -1 **do**
  for $j = i+1$ to length($\mathbf{T}$) **do**
    if $T_j - T_i > minsize$ and $T_j - T_i < maxsize$ **then**
      Add TAD (T_i, T_j) to C
n = length(C)
R=null //Initialization of interaction frequency vector
for $p = 1$ to m **do**
  for $q = 1$ to p **do**
    Add IF (p, q) to R
for $r = 1$ to $repeat$ **do**
  Random_shuffle(R) //Random samples fed into online learning solver
  Initialize $\mathbf{X}' \in \mathbb{R}^{1\times n}, \mathbf{Y}' \in \mathbb{R}^{1\times k}, \mathbf{w} \in \mathbb{R}^{1\times n}, \mathbf{z} \in \mathbb{R}^{1\times n}, \mathbf{b} \in \mathbb{R}^{1\times n}$ with all zeros // Initialization of FTRL
  for $it = 1$ to $iteration$ **do**
    for $s = 1$ to length(R) **do**
      Let u = R_s.first , v = R_s.second
      for $j = 1$ to n **do**
        if (u, v) falls into C_j **then**

$$X'_j = \log\left(\frac{m}{|u-v|+1}\right)$$

      Based on designed vector $\mathbf{X}'$, Let $J = \{ j \mid X'_j \neq 0 \}$
      for $i = 1$ to k **do**
        $Y'_i = A_{iuv}$
        for all $j \in J$ **do** //Starting of FTRL

$$b_j = \begin{cases} 0 & \text{if } |z_j| \le l \\ -\left(\dfrac{\beta + \sqrt{w_j}}{\alpha}\right)^{-1} \left(z_j - \operatorname{sgn}(z_j) l\right) & \text{otherwise} \end{cases}$$

        Predict $p_i = \sum_{j \in J} X'_j \operatorname{sgn}(b_j) b_j$ using the b_j computed above
        for all $j \in J$ **do**

$$g_j = 2\operatorname{sgn}(b_j)(p_i - Y'_i) X'_j$$

$$\sigma_j = \frac{1}{\alpha}\left(\sqrt{w_j + g_j^2} - \sqrt{w_j}\right)$$

$$z_j \leftarrow z_j + g_j - \sigma_j b_j$$

$$w_j \leftarrow w_j + g_j^2$$

$\mathbf{b}_r = \mathbf{b}$

$\mathbf{B} = (\mathbf{b}_1^{\mathrm{T}}, \mathbf{b}_2^{\mathrm{T}}, ..., \mathbf{b}_{\mathrm{repeat}}^{\mathrm{T}})$ //Output of regression coefficients

C_s = { C_i | Hierarchical TAD C_i is significant}

B_s = { B_i | B_i is for a hierarchical TAD in C_s }

Return C_s and B_s

Supplementary Note 2 Configurations of TADfit and competing methods

TADfit. Our TADfit is a multivariate linear regression model for profiling hierarchical TADs on replicate Hi-C data, which tries to fit the interaction frequencies in Hi-C contact matrix with and without replicates using all-possible hierarchical TADs, and the significant ones can be determined by the regression coefficients obtained with the help of an online learning solver FTRL. To implement this method, the source code can be downloaded from https://github.com/lhqxinghun/TADfit, and there are eight parameters need to be tuned. During the implementation of TADfit in this study, we configured these parameters by default: winsize = 5, maxsize = 200 bins, minsize = 3 bins, repeat = 5, iteration = 2, α = 0.01, β = 1, l = 2. For segmented contact matrices, maxsize and minsize were set to 0.50 and 0.01 times the length of the segment, respectively, leaving the other parameters as default values.

TADtree. TADtree is the first publicly available method to identify hierarchical TADs on Hi-C data [3], which formulates and optimizes an objective function to score a hierarchy of nested TAD trees according to both the fit to the observed contact matrix and the boundary index of each TAD and sub-TAD in the hierarchy. To implement this method, the source code was downloaded from http://compbio.cs.brown.edu/projects/tadtree/, and there are six parameters need to be tuned. According to the example control file given by the original proposer, we configured these parameters as follows: p = 3, q = 12 (boundary index parameter), gamma = 500 (balance between boundary index and squared error in score function), S = 200 (maximum size of TAD measured in bins), M = 25 (maximum number of TADs in each TAD tree) and N (maximum number of TADs called) is determined by 6 TADs/Mb.

3DNetMod. 3DNetMod treats the TAD identification as a community detection problem in graph theory [4]. This method identifies the nested, partially overlapping TADs and subTADs by optimizing network modularity through a Louvain-like locally greedy algorithm. To implement this method, the source code was downloaded from https://bitbucket.org/creminslab/3dnetmod_method_v1.0_10_06_17/, and there are a number of parameters need to be tuned. According to the recommendation of the original proposer, we configured these parameters as follows: region_size = 200 (measured in bins), overlap = 100 (25Kb and 50 Kb) or 200 (10Kb), logged = True, badregionfilter = True, plateau = 3, chaosfilter = True, diagonal_density = 0.95, num_part = 20, plots = False, pctile_threshold = 0, pct_value = 0, size_threshold = 4, size_s1 = 400000, size_s2 = 800000, size_s3 = 1600000, size_s4 = 3000000, size_s5 = 12000000, boundary_buffer = resolution of the input Hi-C contact matrix.

OnTAD. OnTAD identifies nested TADs by a two-step approach [5]. It first detects the candidate TAD boundaries by a modified TopDom method, and then tries to assemble these candidate boundaries into a hierarchical TADs structure optimized by a recursive dynamic programming algorithm. The program of OnTAD (v. 1.2) was downloaded from https://github.com/anlin00007/OnTAD/. To implement this method, there are seven parameters required to be tuned. During the implementation of OnTAD, we configured these parameters as default: maxsz = 200 (maximum size of called TADs measured in bins), minsz = 3 (minimum size of called TADs), penalty = 0.1 (penalty in optimal function to select positive TADs), ldiff = 1.96 (cut-off to determine local minumum), lsize =5 (local region size that used to determine local minimum), bedout = False (specify the format of output file, this parameter is required when input .hic file) and hic_norm = None (normalization method).

SpectralTAD. SpectralTAD identifies hierarchical TADs by a modified version of the multiclass spectral clustering algorithm [6]. The initial TADs are obtained by maximizing an average silhouette score, and the hierarchical structures are

then determined by iteratively portioning the initial TADs. This method was implemented using R package SpectralTAD (v. 1.2.0) which can be downloaded from BiocManager. During the implementation, the levels of hierarchical structures was set to 3 according to the recommendation of original proposer in their paper. The parameter qual_filter was set to False, and min_size was set to 5 by default.

TADpole. TADpole combines prominent component analysis (PCA) and hierarchical clustering to investigate the continuous nested hierarchical segmentation of Hi-C contact matrix [7]. It was implemented with R package TADpole (v.0.0.0.9) which is available at https://github.com/3DGenomes/TADpole/. To implement this method, there are two parameters need to be tuned. According to the recommendation of the original proposer, we configured these parameters as follows: max_pcs = 200 (maximum number of prominent component) and centromere_search = False (whether to split the chromosome into two sub-matrices by centromere).

II. Supplementary Figures

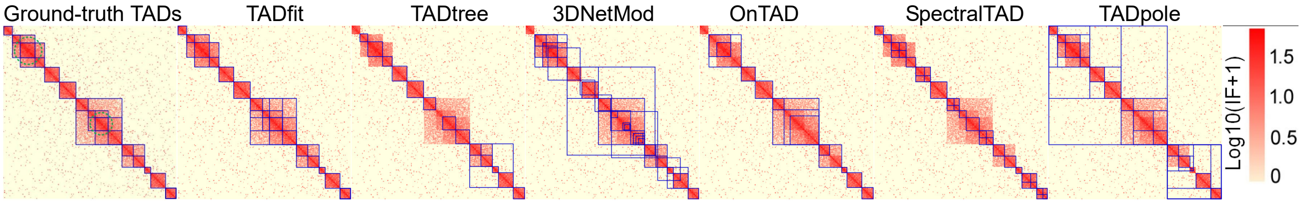

Supplementary Fig. 1 Heatmaps of a simulated contact matrix (ChrS_MAT_noise0.04_POP0.15_rep1, bin 1–bin 200 out from a total of about 400 bins) and the hierarchical TADs called by TADfit and the other five methods on it. A noise of 4% level and partially overlapping TADs were both considered in the simulated contact matrix. The ground-truth TADs as well as the TADs called by these different methods were outlined with blue solid lines, and the partially overlapping TADs were marked with a green dotted circle on the first heatmap.

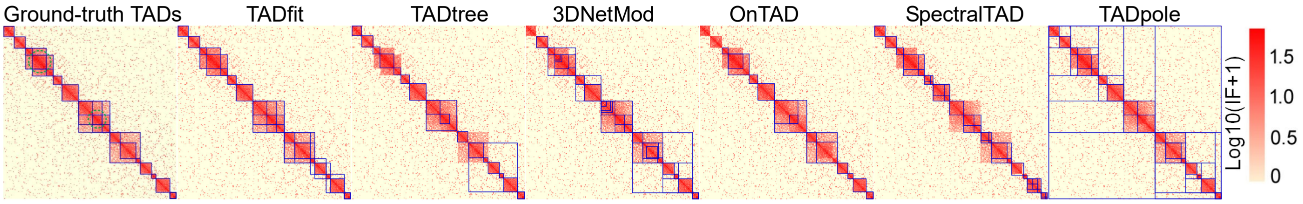

Supplementary Fig. 2 Heatmaps of a simulated contact matrix (ChrS_MAT_noise0.08_POP0.15_rep1, bin 1–bin 200 out from a total of about 400 bins) and the hierarchical TADs called by TADfit and the other five methods on it. A noise of 8% level and partially overlapping TADs were both considered in the simulated contact matrix. The ground-truth TADs as well as the TADs called by these different methods were outlined with blue solid lines, and the partially overlapping TADs were marked with a green dotted circle on the first heatmap.

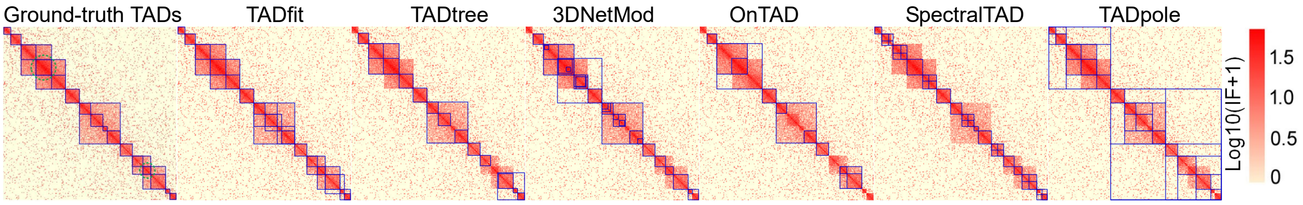

Supplementary Fig. 3 Heatmaps of a simulated contact matrix (ChrS_MAT_noise0.12_POP0.15_rep1, bin 1–bin 200 out from a total of about 400 bins) and the hierarchical TADs called by TADfit and the other five methods on it. A noise of 12% level and partially overlapping TADs were both considered in the simulated contact matrix. The ground-truth TADs as well as the TADs called by these different methods were outlined with blue solid lines, and the partially overlapping TADs were marked with a green dotted circle on the first heatmap.

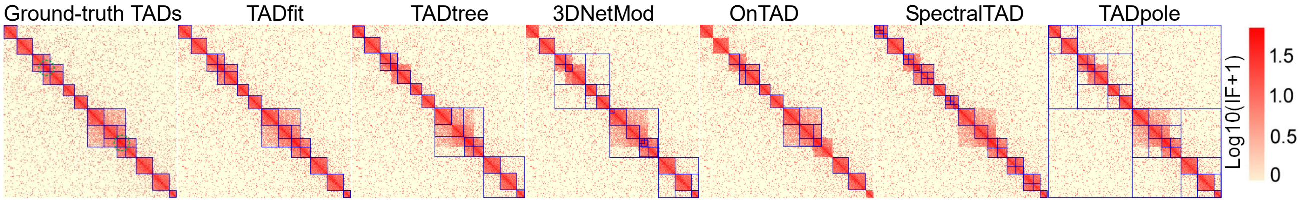

Supplementary Fig. 4 Heatmaps of a simulated contact matrix (ChrS_MAT_noise0.16_POP0.15_rep1, bin 1–bin 200 out from a total of about 400 bins) and the hierarchical TADs called by TADfit and the other five methods on it. A noise of 16% level and partially overlapping TADs were both considered in the simulated contact matrix. The ground-truth TADs as well as the TADs called by these different methods were outlined with blue solid lines, and the partially overlapping TADs were marked with a green dotted circle on the first heatmap.

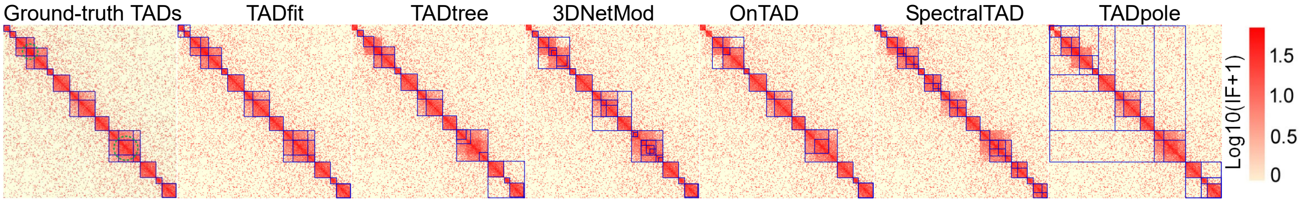

Supplementary Fig. 5 Heatmaps of a simulated contact matrix (ChrS_MAT_noise0.20_POP0.15_rep1, bin 1–bin 200 out from a total of about 400 bins) and the hierarchical TADs called by TADfit and the other five methods on it. A noise of 20% level and partially overlapping TADs were both considered in the simulated contact matrix. The ground-truth TADs as well as the TADs called by these different methods were outlined with blue solid lines, and the partially overlapping TADs were marked with a green dotted circle on the first heatmap.

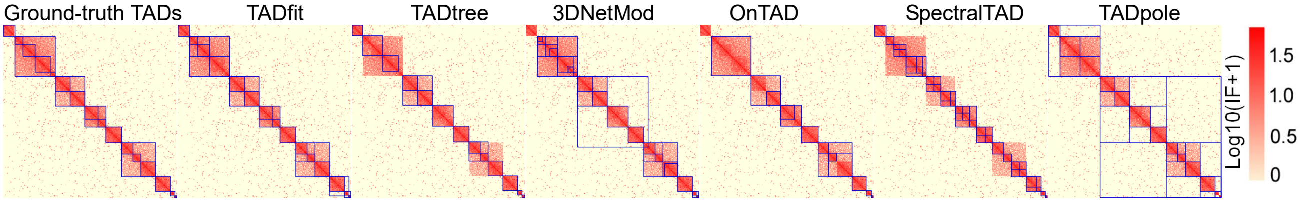

Supplementary Fig. 6 Heatmaps of a simulated contact matrix (ChrS_MAT_noise0.04_POP0 _rep1, bin 1–bin 200 out from a total of about 400 bins) and the hierarchical TADs called by TADfit and the other five methods on it. A noise of 4% level was considered in the simulated contact matrix, but partially overlapping TADs were not. The ground-truth TADs as well as the TADs called by these different methods were outlined with blue solid lines.

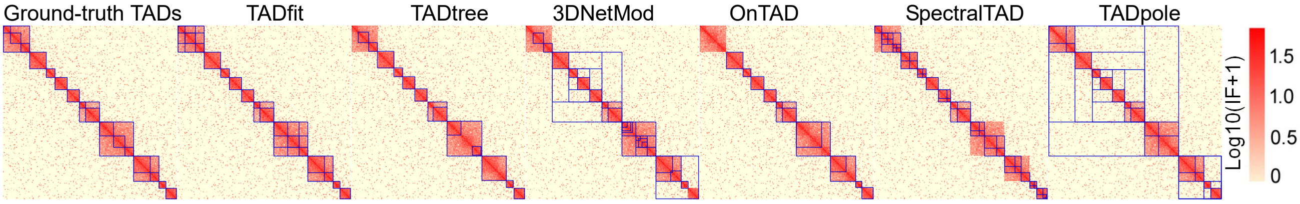

Supplementary Fig. 7 Heatmaps of a simulated contact matrix (ChrS_MAT_noise0.08_POP0 _rep1, bin 1–bin 200 out from a total of about 400 bins) and the hierarchical TADs called by TADfit and the other five methods on it. A noise of 8% level was considered in the simulated contact matrix, but partially overlapping TADs were not. The ground-truth TADs as well as the TADs called by these different methods were outlined with blue solid lines.

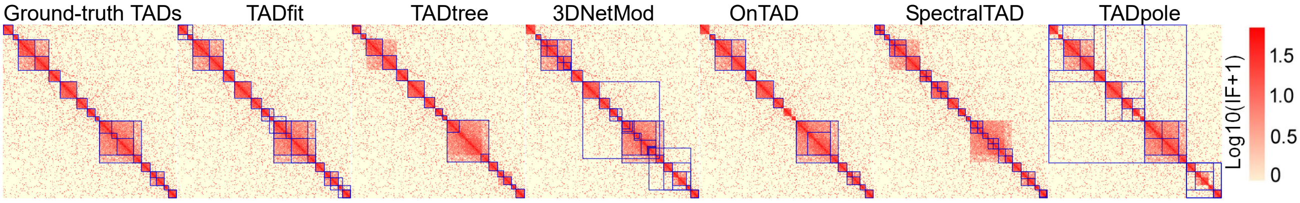

Supplementary Fig. 8 Heatmaps of a simulated contact matrix (ChrS_MAT_noise0.12_POP0 _rep1, bin 1–bin 200 out from a total of about 400 bins) and the hierarchical TADs called by TADfit and the other five methods on it. A noise of 12% level was considered in the simulated contact matrix, but partially overlapping TADs were not. The ground-truth TADs as well as the TADs called by these different methods were outlined with blue solid lines.

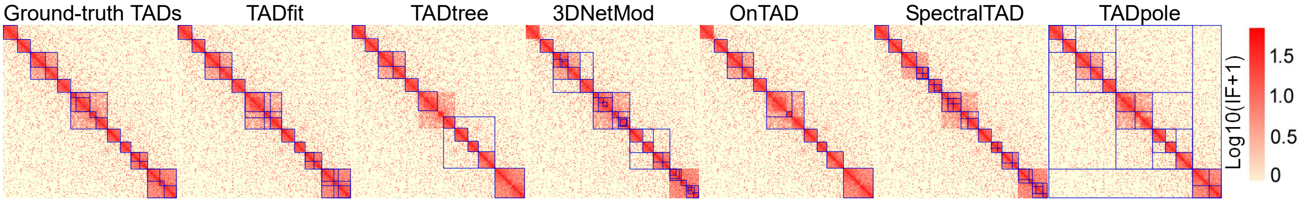

Supplementary Fig. 9 Heatmaps of a simulated contact matrix (ChrS_MAT_noise0.16_POP0 _rep1, bin 1–bin 200 out from a total of about 400 bins) and the hierarchical TADs called by TADfit and the other five methods on it. A noise of 16% level was considered in the simulated contact matrix, but partially overlapping TADs were not. The ground-truth TADs as well as the TADs called by these different methods were outlined with blue solid lines.

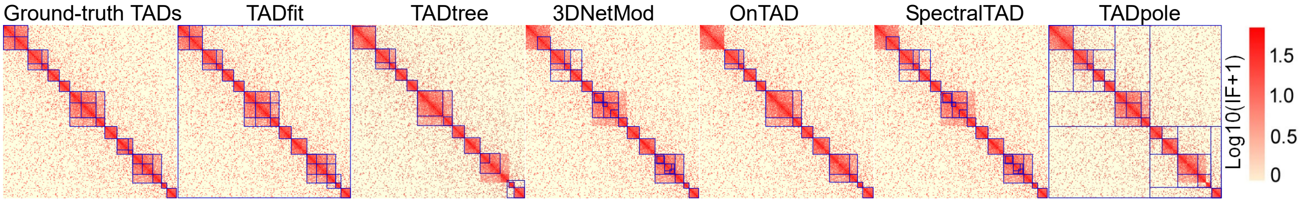

Supplementary Fig. 10 Heatmaps of a simulated contact matrix (ChrS_MAT_noise0.20_POP0 _rep1, bin 1–bin 200 out from a total of about 400 bins) and the hierarchical TADs called by TADfit and the other five methods on it. A noise of 20% level was considered in the simulated contact matrix, but partially overlapping TADs were not. The ground-truth TADs as well as the TADs called by these different methods were outlined with blue solid lines.

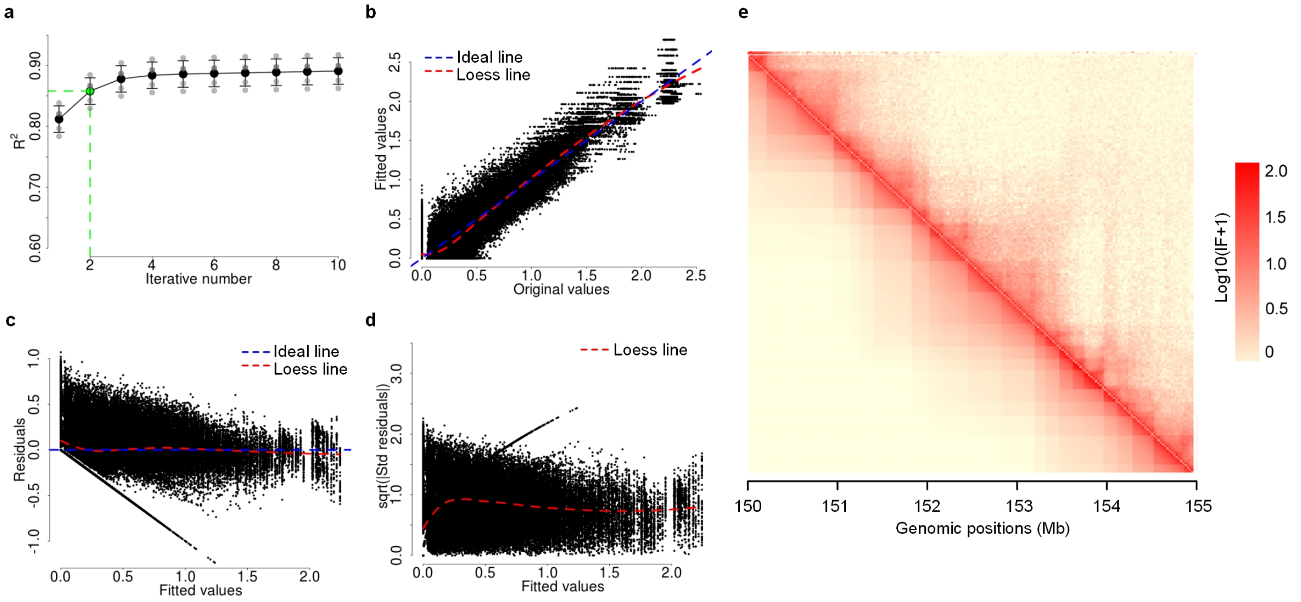

Supplementary Fig. 11 Regression analysis of the proposed model TADfit on contact matrix replicates (GSM1551550_HIC001–GSM1551554_HIC005) for chromosome 1 of GM12878 at 25K resolution. a A curve of R^2 (mean ± SD) versus iterative number, as well as a total of three scatter plots, including **b** plot of fitted values versus original values, **c** plot of residuals versus fitted values and **d** scale location plot, were plotted, and the loess-fitted curves were drawn with red dotted line. Besides, **e** the heatmap of an artificial contact matrix with the upper right triangle for original values (GSM1551550_HIC001, 150 Mb–155 Mb) and the lower left triangle for the corresponding fitted values was shown.

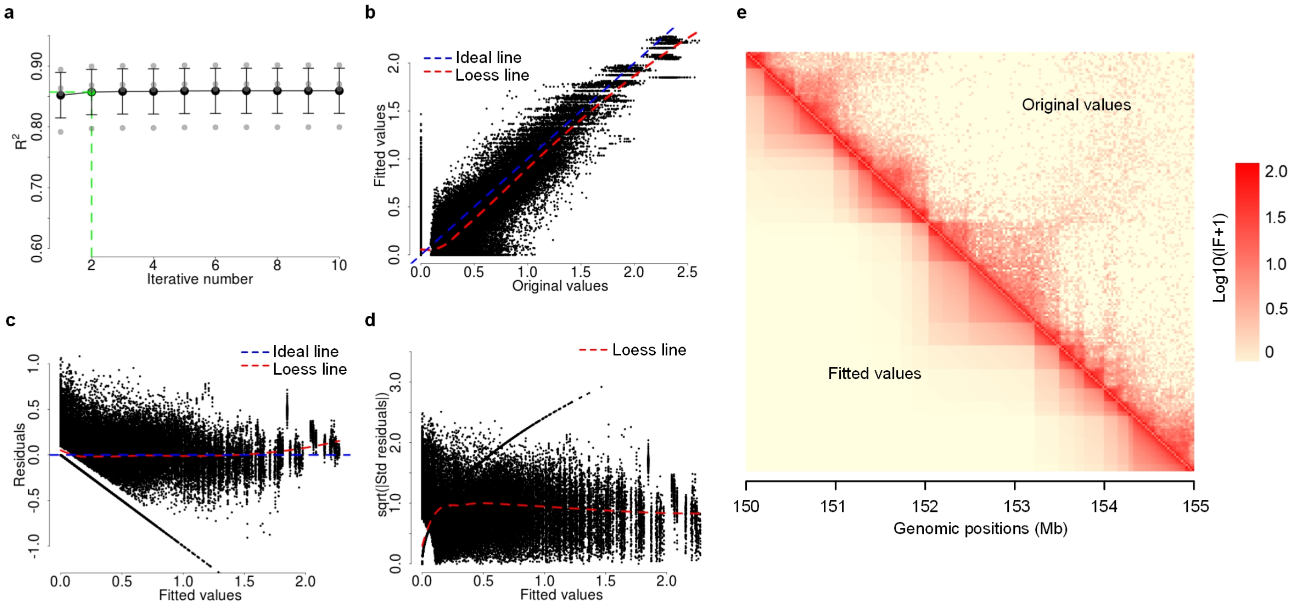

Supplementary Fig. 12 Regression analysis of the proposed model TADfit on contact matrix replicates (GSM1551599_HIC050–GSM1551604_HIC055) for chromosome 1 of IMR90 at 25K resolution. a A curve of R^2 (mean ± SD) versus iterative number, as well as a total of three scatter plots, including **b** plot of fitted values versus original values, **c** plot of residuals versus fitted values and **d** scale location plot, were plotted, and the loess-fitted curves were drawn with red dotted line. Besides, **e** the heatmap of an artificial contact matrix with the upper right triangle for original values (GSM1551599_HIC050, 150 Mb–155 Mb) and the lower left triangle for the corresponding fitted values was shown.

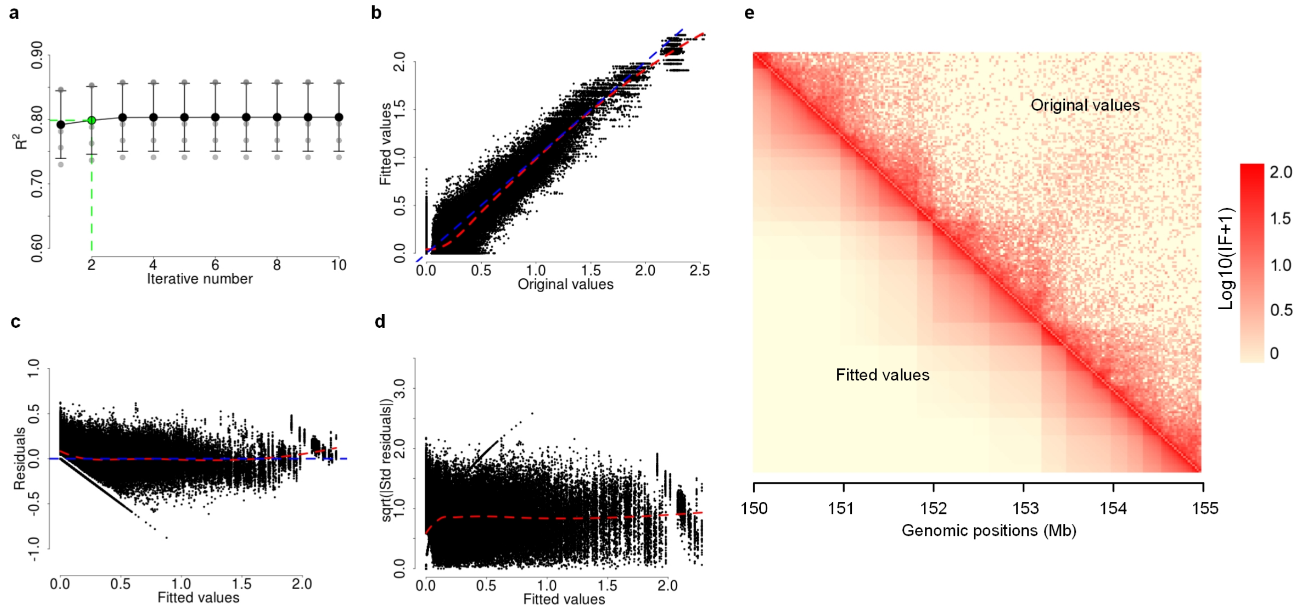

Supplementary Fig. 13 Regression analysis of the proposed model TADfit on contact matrix replicates (GSM1551619_HIC070–GSM1551623_HIC074) for chromosome 1 of K562 at 25K resolution. **a** A curve of R^2 (mean ± SD) versus iterative number, as well as a total of three scatter plots, including **b** plot of fitted values versus original values, **c** plot of residuals versus fitted values and **d** scale location plot, were plotted, and the loess-fitted curves were drawn with red dotted line. Besides, **e** the heatmap of an artificial contact matrix with the upper right triangle for original values (GSM1551619_HIC070, 150 Mb–155 Mb) and the lower left triangle for the corresponding fitted values was shown.

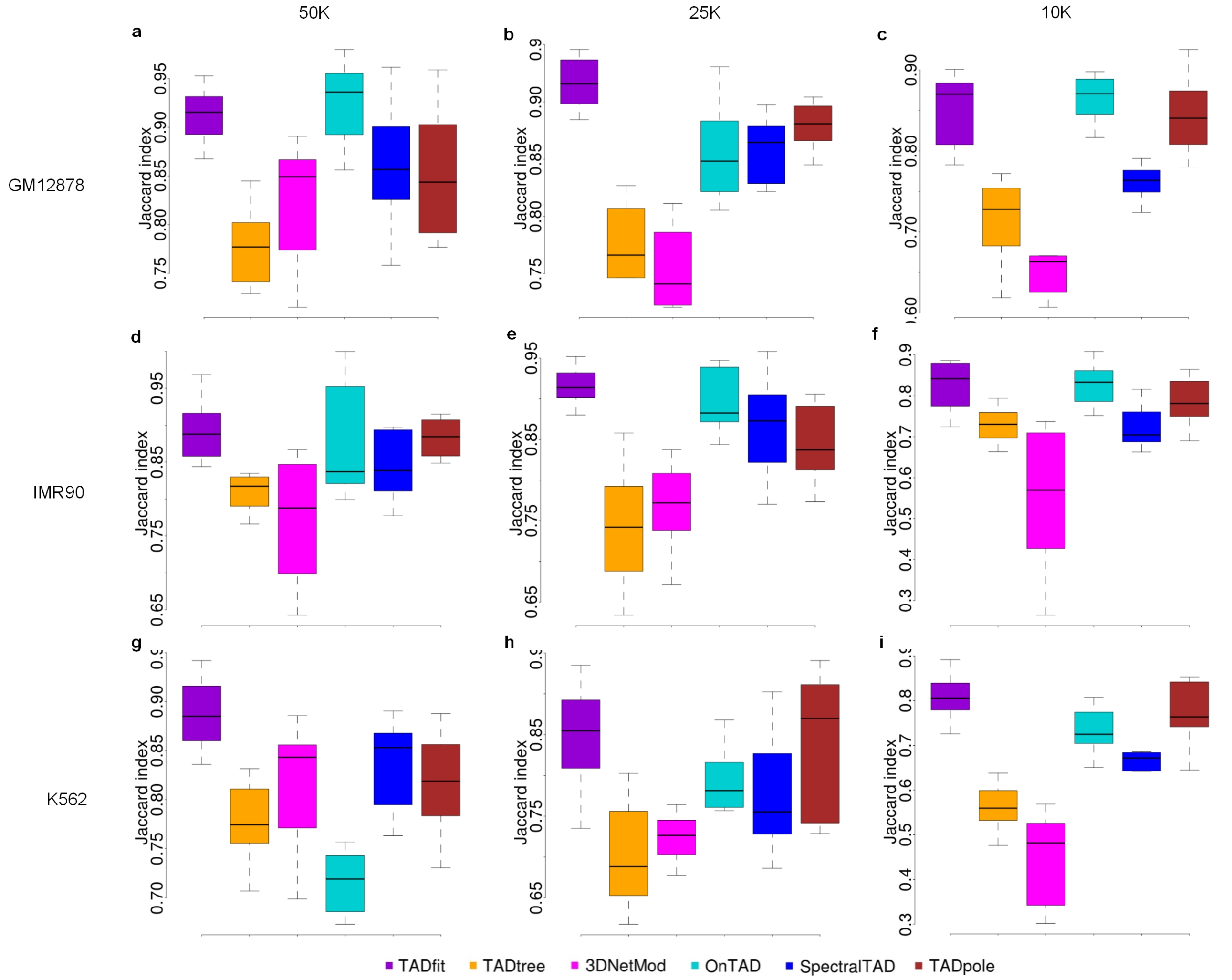

Supplementary Fig. 14 Reproducibility of hierarchical TADs called by TADfit and the other five methods across replicates. The hierarchical TADs were called on contact matrix replicates (GSM1551550_HIC001–GSM1551554_HIC005) for chromosome 1 of GM12878 at resolutions of **a** 50K, **b** 25K and **c** 10K, on contact matrix replicates (GSM1551599_HIC050–GSM1551604_HIC055) for chromosome 1 of IMR90 at resolutions of **d** 50K, **e** 25K and **f** 10K, and on contact matrix replicates (GSM1551619_HIC070–GSM1551623_HIC074) for chromosome 1 of K562 at resolutions of **g** 50K, **h** 25K and **i** 10K, respectively. To conduct a comparative analysis on an equal footing, the contact matrix replicates were fed into TADfit individually, since TADfit is a multi-replicate method, the Jaccard index of hierarchical TADs called by it between replicates should always be one, which is always higher than those of the other methods where a multi-replicate input is not allowed. The center line of the box indicates the median, whereas the bottom and top of the box indicate the first and third quartiles, respectively, and whiskers are extended to the most extreme data point that is no more than 1.5 × interquartile range from the bottom and top of the box.

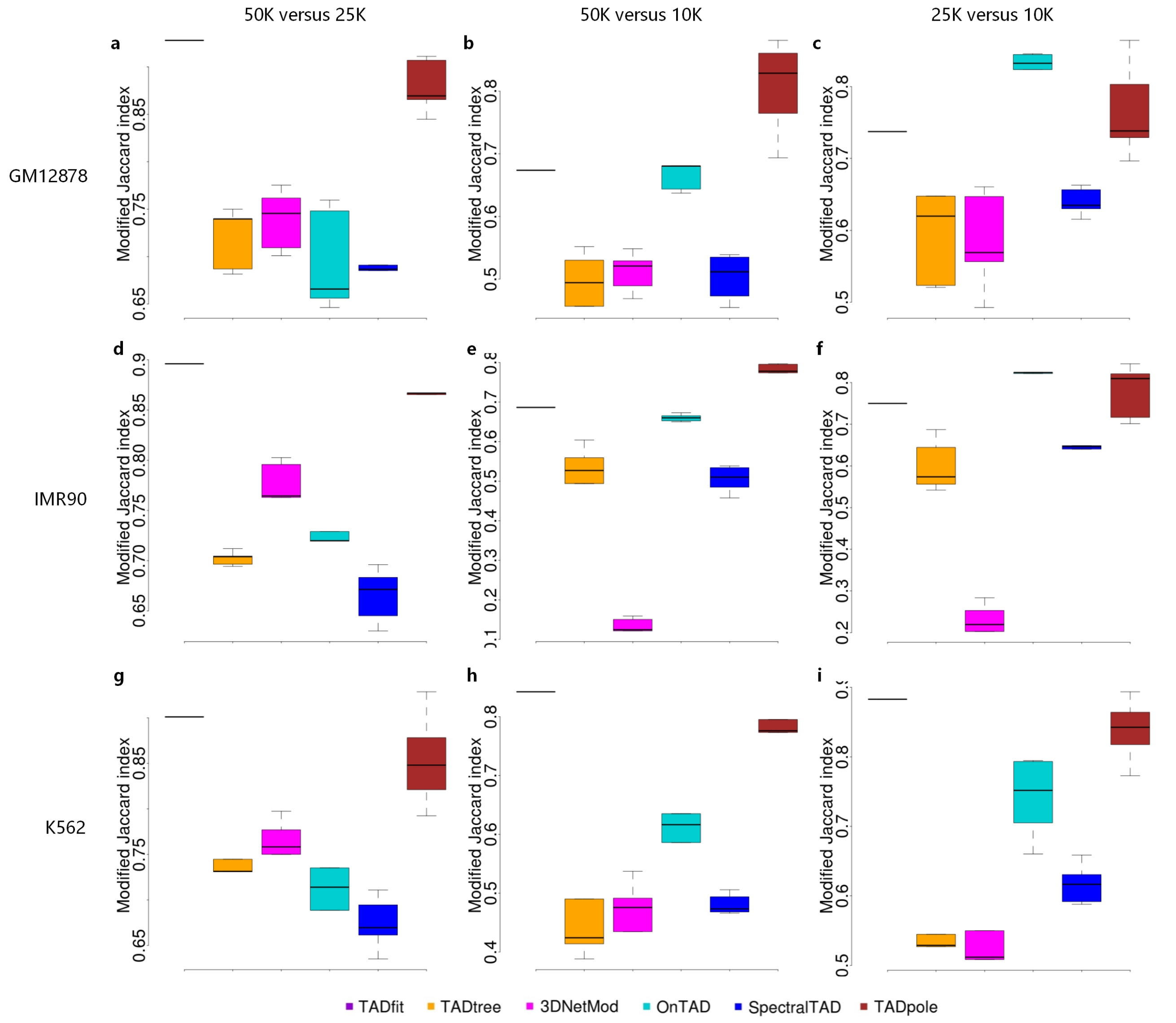

Supplementary Fig. 15 Reproducibility of hierarchical TADs called by TADfit and the other five methods between different resolutions. The hierarchical TADs were called on contact matrix replicates (GSM1551550_HIC001–GSM1551554_HIC005) for chromosome 1 of GM12878 between resolutions of **a** 50K and 25K, **b** 50K and 10K, as well as **c** 25K and 10K, on contact matrix replicates (GSM1551599_HIC050–GSM1551604_HIC055) for chromosome 1 of IMR90 between resolutions of **d** 50K and 25K, **e** 50K and 10K, as well as **f** 25K and 10K, and on contact matrix replicates (GSM1551619_HIC070–GSM1551623_HIC074) for chromosome 1 of K562 between resolutions of **g** 50K and 25K, **h** 50K and 10K, as well as **i** 25K and 10K, respectively. The center line of the box indicates the median, whereas the bottom and top of the box indicate the first and third quartiles, respectively, and whiskers are extended to the most extreme data point that is no more than 1.5 × interquartile range from the bottom and top of the box.

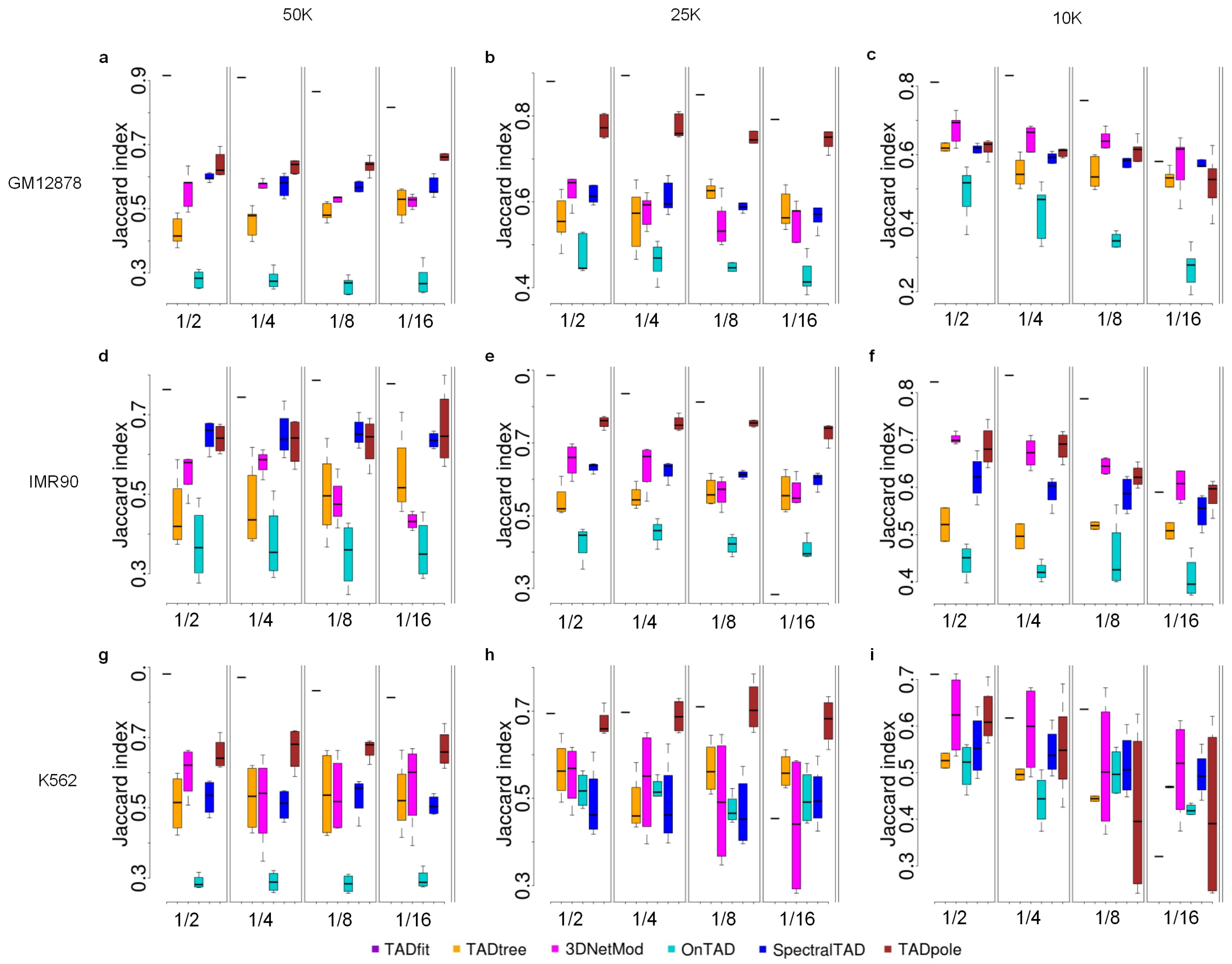

Supplementary Fig. 16 Variation of hierarchical TADs called by TADfit and the other five methods at different sequencing depths (1/2, 1/4, 1/8 and 1/16). The hierarchical TADs were called on contact matrix replicates (GSM1551550_HIC001–GSM1551550_HIC005) for chromosome 1 of GM12878 at resolutions of **a** 50K, **b** 25K and **c** 10K, on contact matrix replicates (GSM1551599_HIC050–GSM1551604_HIC055) for chromosome 1 of IMR90 at resolutions of **d** 50K, **e** 25K and **f** 10K, and on contact matrix replicates (GSM1551619_HIC070–GSM1551623_HIC074) for chromosome 1 of K562 at resolutions of **g** 50K, **h** 25K and **i** 10K, respectively. The center line of the box indicates the median, whereas the bottom and top of the box indicate the first and third quartiles, respectively, and whiskers are extended to the most extreme data point that is no more than 1.5 × interquartile range from the bottom and top of the box.

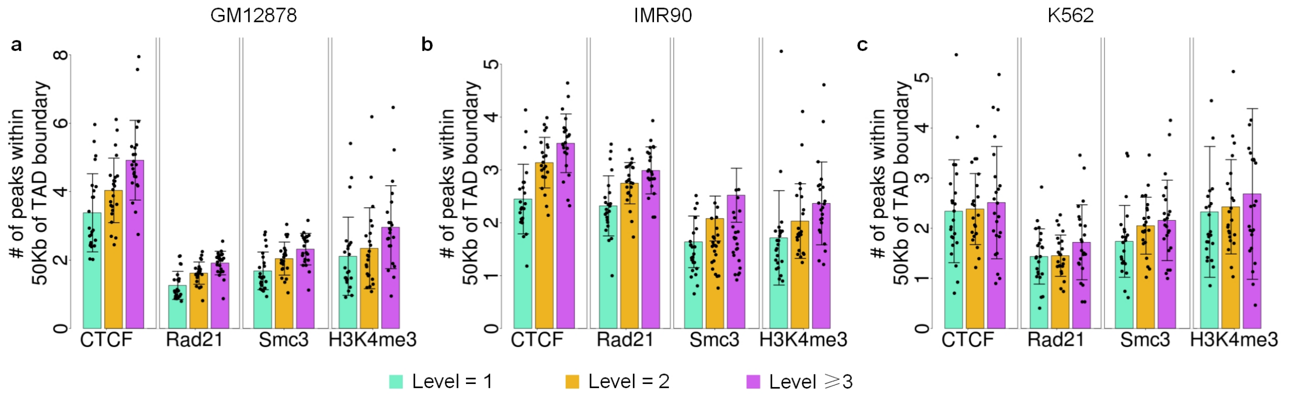

Supplementary Fig. 17 Enrichment of architectural proteins and histone mark within 50 Kb of multiple-level boundaries of hierarchical TADs called by TADfit on a genome-wide scale. The hierarchical TADs were called **a** on contact matrix replicates (GSM1551550_HIC001–GSM1551554_HIC005) for all chromosomes of GM12878, **b** on contact matrix replicates (GSM1551599_HIC050–GSM1551604_HIC055) for all chromosomes of IMR90, and **c** on contact matrix replicates (GSM1551619_HIC070–GSM1551623_HIC074) for all chromosomes of K562 at 25K resolution, respectively. The level of a TAD boundary is defined using the terminology introduced by An, L. et al. [5], that is, a TAD boundary belonging to a single TAD is regarded as a first-level boundary, and the second-level and third-level boundaries correspond to the boundaries that are shared by two and three hierarchical TADs, respectively. The peak files of ChIP-seq for architectural proteins (CTCF, Smc3 and Rad21) and histone mark (H3K4me3) were downloaded from ENCODE (Supplementary Table 3), and the number (mean ± SD) of ChIP-seq peaks within 50 Kb of TAD boundaries at three different levels was calculated.

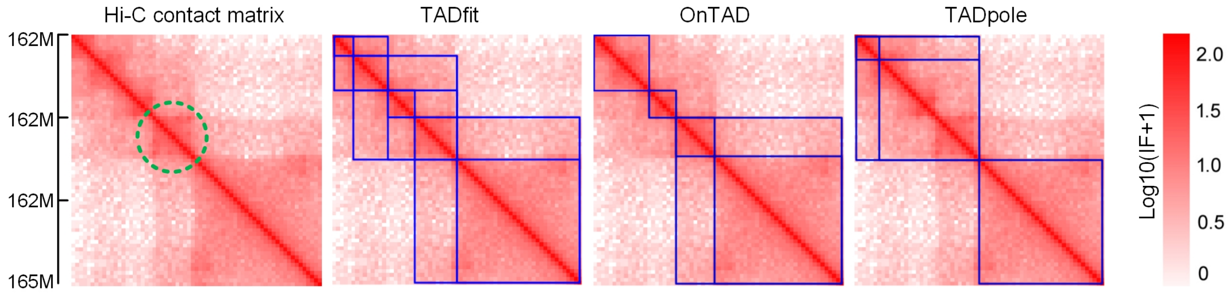

Supplementary Fig. 18 Comparison of the hierarchical TADs called by TADfit, OnTAD and TADpole on contact matrices at 50K resolution, especially in handling partially overlapping TADs. The hierarchical TADs were called by TADfit on contact matrix replicates (GSM1551550_HIC001–GSM1551550_HIC005) for chromosome 1 of GM12878 at 50K resolution, and one of the replicates (GSM1551550_HIC001) was fed into OnTAD and TADpole, since they cannot accept multiple replicates as input. There are four heatmaps drawn above (GSM1551550_HIC001, 162 Mb–165 Mb), on the first of which a partially overlapping TAD was marked with a green dotted circle, and on the last three of which the called TADs were outlined with blue solid line.

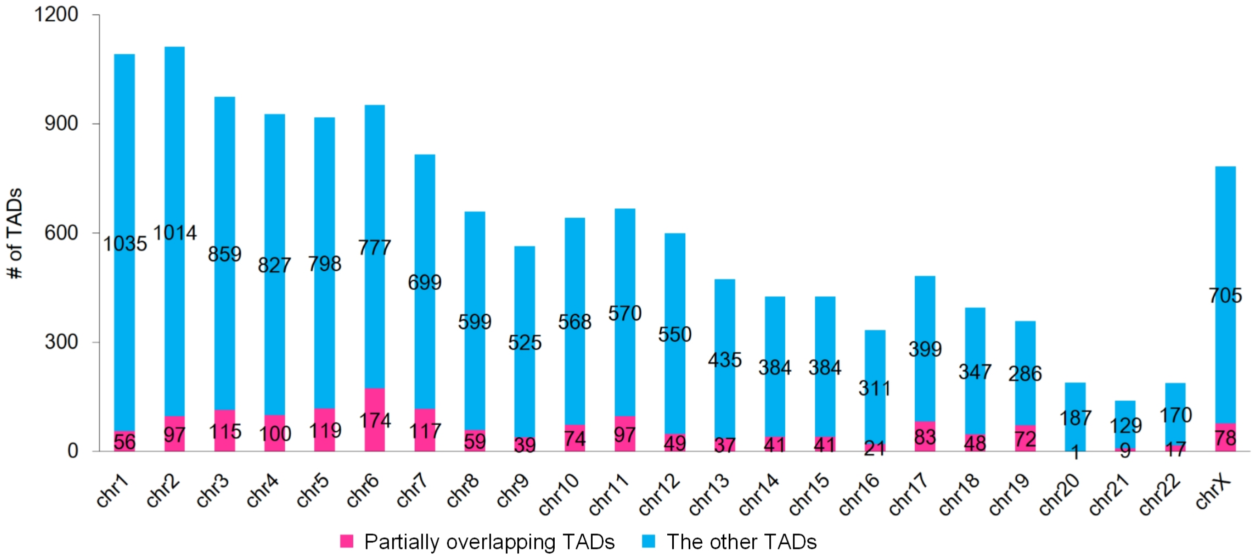

Supplementary Fig. 19 Numbers of partially overlapping TADs and the other TADs called by TADfit on a genome-wide scale. The hierarchical TADs were called on contact matrix replicates (GSM1551550_HIC001–GSM1551554_HIC005) for all chromosomes of GM12878 at resolution of 25K.

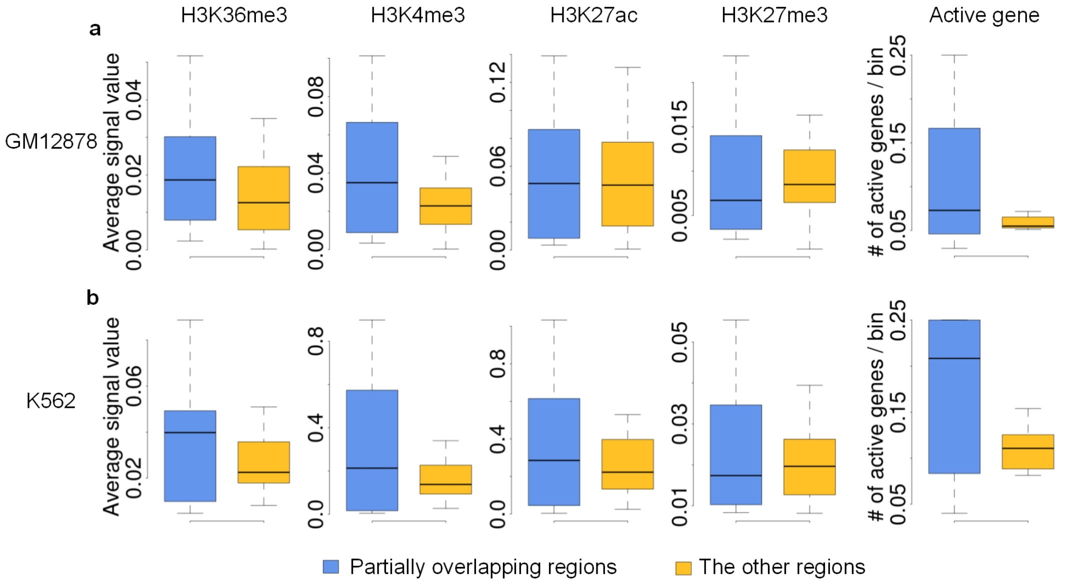

Supplementary Fig. 20 Comparison of biological features between partially overlapping regions and the other regions of hierarchical TADS called by TADfit. The hierarchical TADs were called **a** on contact matrix replicates (GSM1551550_HIC001–GSM1551554_HIC005) for chromosome 1 of GM12878, and **b** on contact matrix replicates (GSM1551619_HIC070–GSM1551623_HIC074) for chromosome 1 of K562 at 25K resolution, respectively. The .bigwig files of ChIP-seq for histone marks (H3K36me3, H3K4me3, H3K27ac and H3K27me3) and the .tsv files for RNA-seq data were downloaded from ENCODE (Supplementary Table 3). The average ChIP-seq signals as well as the density of active genes (FPKM > 5) within both partially overlapping regions and the other regions of hierarchical TADs were shown. The center line of the box indicates the median, whereas the bottom and top of the box indicate the first and third quartiles, respectively, and whiskers are extended to the most extreme data point that is no more than 1.5 × interquartile range from the bottom and top of the box.

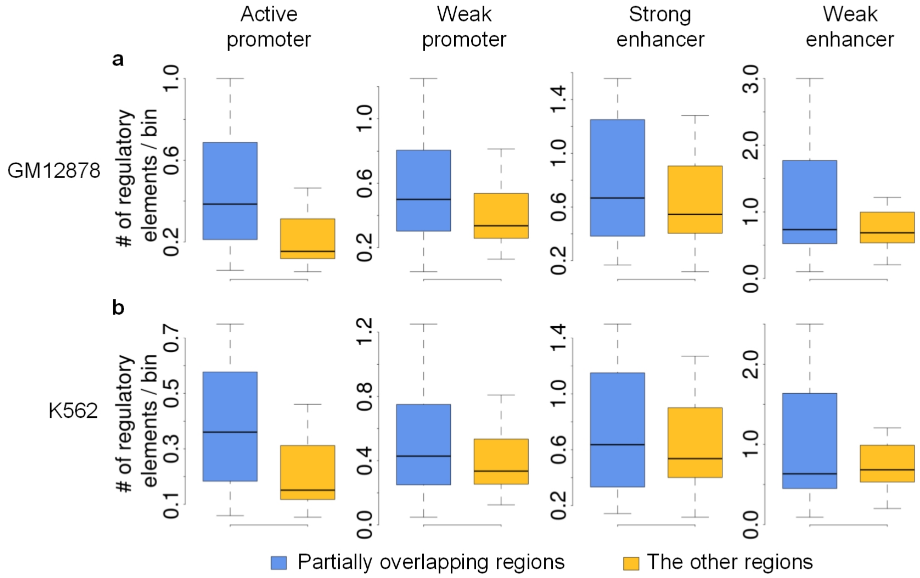

Supplementary Fig. 21 Comparison of regulatory elements between partially overlapping regions and the other regions of hierarchical TADS called by TADfit. The hierarchical TADs were called **a** on contact matrix replicates (GSM1551550_HIC001–GSM1551554_HIC005) for chromosome 1 of GM12878, and **b** on contact matrix replicates (GSM1551619_HIC070–GSM1551623_HIC074) for chromosome 1 of K562 at 25K resolution, respectively. The .bed files for genomic annotations were downloaded from UCSC genome browser (Supplementary Table 3). The density of four categories of regulatory elements (active promoter, weak promoter, strong enhancer and weak enhancer) within both partially overlapping regions and the other regions of hierarchical TADs was shown. The center line of the box indicates the median, whereas the bottom and top of the box indicate the first and third quartiles, respectively, and whiskers are extended to the most extreme data point that is no more than 1.5 × interquartile range from the bottom and top of the box.

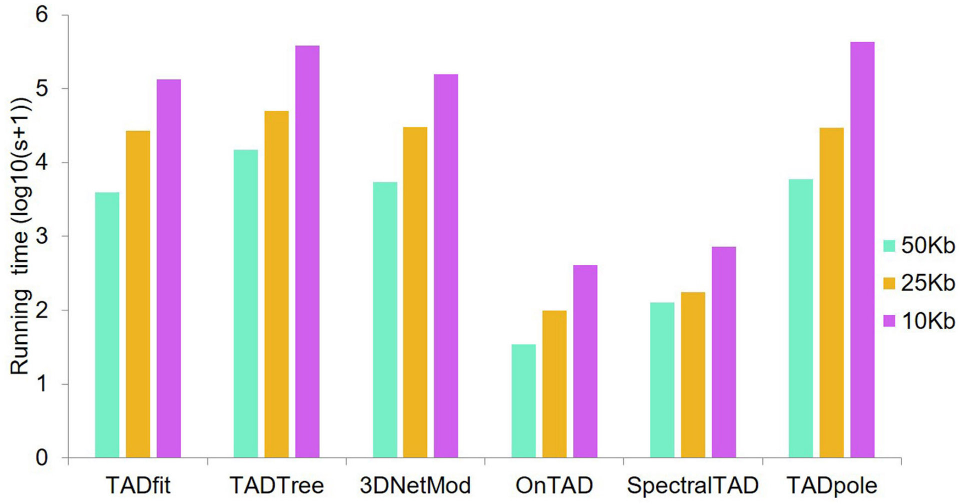

Supplementary Fig. 22 Comparison of running time between TADfit and the other five hierarchical TADs callers, including TADtree, 3DNetMod, OnTAD, SpectralTAD and TADpole, on the same computing platform. The comparison was conducted on contact matrix replicates (GSM1551599_HIC050–GSM1551604_HIC055) for the whole chromosome 8 of IMR90 at resolutions of 50K, 25K and 10K, and the corresponding dimensions of input contact matrices are 2920×2920, 5840×5840 and 14600×14600, respectively. The contact matrix replicates at the same resolution level were all fed into TADfit at one time, but into the other five callers individually, since only TADfit is a multi-replicate method.

III. Supplementary Tables

Supplementary Table 1 Accuracy of the proposed TADfit by Jaccard index on simulated Hi-C data in different cases.

Noise level	With partially overlapping TADs			Without partially overlapping TADs		
	Individual sample	Average value for individual sample	Multiple replicates	Individual sample	Average value for individual sample	Multiple replicates
4%	0.97	0.97 ± 0.01	**0.98**	0.94	0.93 ± 4.47e-3	**0.95**
	0.98			0.93		
	0.95			0.93		
	0.98			0.93		
	0.98			0.93		
8%	0.93	0.95 ± 0.01	**0.96**	0.95	0.94 ± 0.01	**0.98**
	0.95			0.95		
	0.95			0.95		
	0.94			0.94		
	0.95			0.93		
12%	0.91	0.92 ± 0.01	**0.93**	0.87	0.90 ± 0.02	**0.93**
	0.93			0.90		
	0.91			0.90		
	0.94			0.91		
	0.91			0.93		
16%	0.94	0.95 ± 0.01	**0.95**	0.94	0.93 ± 0.01	**0.95**
	0.95			0.93		
	0.95			0.92		
	0.95			0.93		
	0.94			0.94		
20%	0.91	0.93 ± 0.01	**0.94**	0.92	0.92 ± 4.47e-3	**0.95**
	0.93			0.91		
	0.91			0.92		
	0.93			0.92		
	0.92			0.92		

Supplementary Table 2 Accuracy of the proposed TADfit by F1 score on simulated Hi-C data in different cases.

Noise level	With partially overlapping TADs			Without partially overlapping TADs		
	Individual sample	Average value for individual sample	Multiple replicates	Individual sample	Average value for individual sample	Multiple replicates
4%	0.90	0.91 ± 0.03	**0.95**	0.83	0.83 ± 0.01	**0.88**
	0.95			0.83		
	0.86			0.84		
	0.92			0.84		
	0.93			0.84		
8%	0.81	0.85 ± 0.03	**0.88**	0.87	0.86 ± 0.01	**0.93**
	0.87			0.87		
	0.87			0.85		
	0.84			0.86		
	0.86			0.84		
12%	0.78	0.79 ± 0.03	**0.83**	0.71	0.77 ± 0.04	**0.83**
	0.82			0.76		
	0.76			0.77		
	0.83			0.79		
	0.76			0.82		
16%	0.85	0.88 ± 0.02	**0.89**	0.84	0.83 ± 0.02	**0.87**
	0.88			0.83		
	0.90			0.82		
	0.90			0.81		
	0.86			0.86		
20%	0.77	0.78 ± 0.02	**0.83**	0.82	0.82 ± 4.47e-3	**0.89**
	0.80			0.82		
	0.75			0.82		
	0.80			0.83		
	0.79			0.82		

Supplementary Table 3 Simulated Hi-C data involved in the paper.

With partially overlapping hierarchical TADs	Noise level	Replicate
Yes	4%	ChrS_MAT_noise0.04_POP0.15_rep1
Yes	4%	ChrS_MAT_noise0.04_POP0.15_rep2
Yes	4%	ChrS_MAT_noise0.04_POP0.15_rep3
Yes	4%	ChrS_MAT_noise0.04_POP0.15_rep4
Yes	4%	ChrS_MAT_noise0.04_POP0.15_rep5
Yes	8%	ChrS_MAT_noise0.08_POP0.15_rep1
Yes	8%	ChrS_MAT_noise0.08_POP0.15_rep2
Yes	8%	ChrS_MAT_noise0.08_POP0.15_rep3
Yes	8%	ChrS_MAT_noise0.08_POP0.15_rep4
Yes	8%	ChrS_MAT_noise0.08_POP0.15_rep5
Yes	12%	ChrS_MAT_noise0.12_POP0.15_rep1
Yes	12%	ChrS_MAT_noise0.12_POP0.15_rep2
Yes	12%	ChrS_MAT_noise0.12_POP0.15_rep3
Yes	12%	ChrS_MAT_noise0.12_POP0.15_rep4
Yes	12%	ChrS_MAT_noise0.12_POP0.15_rep5
Yes	16%	ChrS_MAT_noise0.16_POP0.15_rep1
Yes	16%	ChrS_MAT_noise0.16_POP0.15_rep2
Yes	16%	ChrS_MAT_noise0.16_POP0.15_rep3
Yes	16%	ChrS_MAT_noise0.16_POP0.15_rep4
Yes	16%	ChrS_MAT_noise0.16_POP0.15_rep5
Yes	20%	ChrS_MAT_noise0.20_POP0.15_rep1
Yes	20%	ChrS_MAT_noise0.20_POP0.15_rep2
Yes	20%	ChrS_MAT_noise0.20_POP0.15_rep3
Yes	20%	ChrS_MAT_noise0.20_POP0.15_rep4
Yes	20%	ChrS_MAT_noise0.20_POP0.15_rep5
No	4%	ChrS_MAT_noise0.04_POP0_rep1
No	4%	ChrS_MAT_noise0.04_POP0_rep2
No	4%	ChrS_MAT_noise0.04_POP0_rep3
No	4%	ChrS_MAT_noise0.04_POP0_rep4
No	4%	ChrS_MAT_noise0.04_POP0_rep5
No	8%	ChrS_MAT_noise0.08_POP0_rep1
No	8%	ChrS_MAT_noise0.08_POP0_rep2
No	8%	ChrS_MAT_noise0.08_POP0_rep3
No	8%	ChrS_MAT_noise0.08_POP0_rep4
No	8%	ChrS_MAT_noise0.08_POP0_rep5
No	12%	ChrS_MAT_noise0.12_POP0_rep1
No	12%	ChrS_MAT_noise0.12_POP0_rep2
No	12%	ChrS_MAT_noise0.12_POP0_rep3
No	12%	ChrS_MAT_noise0.12_POP0_rep4
No	12%	ChrS_MAT_noise0.12_POP0_rep5
No	16%	ChrS_MAT_noise0.16_POP0_rep1
No	16%	ChrS_MAT_noise0.16_POP0_rep2
No	16%	ChrS_MAT_noise0.16_POP0_rep3
No	16%	ChrS_MAT_noise0.16_POP0_rep4
No	16%	ChrS_MAT_noise0.16_POP0_rep5

Supplementary Table 3 Simulated Hi-C data involved in the paper.

With partially overlapping hierarchical TADs	Noise level	Replicate
No	20%	ChrS_MAT_noise0.20_POP0_rep1
No	20%	ChrS_MAT_noise0.20_POP0_rep2
No	20%	ChrS_MAT_noise0.20_POP0_rep3
No	20%	ChrS_MAT_noise0.20_POP0_rep4
No	20%	ChrS_MAT_noise0.20_POP0_rep5

Supplementary Table 4 Experimental Hi-C data involved in the paper

Cell line	Resolution	Replicate	Source	URL for downloading
GM12878	10K/25K/50K	GSM1551550_HIC001	[8]	https://bcm.app.box.com/v/aidenlab/
GM12878	10K/25K/50K	GSM1551551_HIC002	[8]	https://bcm.app.box.com/v/aidenlab/
GM12878	10K/25K/50K	GSM1551552_HIC003	[8]	https://bcm.app.box.com/v/aidenlab/
GM12878	10K/25K/50K	GSM1551553_HIC004	[8]	https://bcm.app.box.com/v/aidenlab/
GM12878	10K/25K/50K	GSM1551554_HIC005	[8]	https://bcm.app.box.com/v/aidenlab/
IMR90	10K/25K/50K	GSM1551599_HIC050	[8,9]	https://bcm.app.box.com/v/aidenlab/
IMR90	10K/25K/50K	GSM1551601_HIC052	[8,9]	https://bcm.app.box.com/v/aidenlab/
IMR90	10K/25K/50K	GSM1551602_HIC053	[8,9]	https://bcm.app.box.com/v/aidenlab/
IMR90	10K/25K/50K	GSM1551603_HIC054	[8,9]	https://bcm.app.box.com/v/aidenlab/
IMR90	10K/25K/50K	GSM1551604_HIC055	[8,9]	https://bcm.app.box.com/v/aidenlab/
K562	10K/25K/50K	GSM1551619_HIC070	[8,10]	https://bcm.app.box.com/v/aidenlab/
K562	10K/25K/50K	GSM1551620_HIC071	[8,10]	https://bcm.app.box.com/v/aidenlab/
K562	10K/25K/50K	GSM1551621_HIC072	[8,10]	https://bcm.app.box.com/v/aidenlab/
K562	10K/25K/50K	GSM1551622_HIC073	[8,10]	https://bcm.app.box.com/v/aidenlab/
K562	10K/25K/50K	GSM1551623_HIC074	[8,10]	https://bcm.app.box.com/v/aidenlab/

Supplementary Table 5 Histone marks, architectural proteins, regulatory elements and RNA-seq data involved in the paper

Cell line	Type	URL for downloading	Accession
GM12878	H3K36me3	https://www.encodeproject.org/experiments/ENCSR000DRW/	ENCFF398BOA
GM12878	H3K4me3	https://www.encodeproject.org/experiments/ENCSR000DRY/	ENCFF166QWE
GM12878	H3K4me3 (peak)	https://www.encodeproject.org/experiments/ENCSR000DRY/	ENCFF621QMO
GM12878	H3K27ac	https://www.encodeproject.org/experiments/ENCSR000AKC/	ENCFF258KTL
GM12878	H3K27me3	https://www.encodeproject.org/experiments/ENCSR000DRX/	ENCFF398JTA
GM12878	CTCF	https://www.encodeproject.org/experiments/ENCSR000DKV/	ENCFF001USH
GM12878	Rad21	https://www.encodeproject.org/experiments/ENCSR000EAC/	ENCFF001VFE
GM12878	Smc3	https://www.encodeproject.org/experiments/ENCSR000DZP/	ENCFF686FLD
GM12878	Promoter& Enhancer	https://hgdownload.soe.ucsc.edu/goldenpath/hg19/encodeDCC/wgEncodeBroadHmm/wgEncodeBroadHmmGm12878HMM.bed.gz	N/A
GM12878	RNA-seq	https://www.encodeproject.org/experiments/ENCSR000AEE/	ENCFF009ZXH
IMR90	H3K4me3 (peak)	https://www.encodeproject.org/experiments/ENCSR087PFU/	ENCFF154CUR
IMR90	CTCF	https://www.encodeproject.org/experiments/ENCSR000EFI/	ENCFF453XKM
IMR90	Rad21	https://www.encodeproject.org/experiments/ENCSR000EFJ/	ENCFF195CYT
IMR90	Smc3	https://www.encodeproject.org/experiments/ENCSR000HPG/	ENCFF770ISZ
K562	H3K36me3	https://www.encodeproject.org/experiments/ENCSR000AKR/	ENCFF464YSK
K562	H3K4me3	https://www.encodeproject.org/experiments/ENCSR000AKU/	ENCFF029DFM
K562	H3K4me3 (peak)	https://www.encodeproject.org/experiments/ENCSR000AKU/	ENCFF127XXD
K562	H3K27ac	https://www.encodeproject.org/experiments/ENCSR000AKP/	ENCFF840LLW
K562	H3K27me3	https://www.encodeproject.org/experiments/ENCSR000AKQ/	ENCFF658JMW
K562	CTCF	https://www.encodeproject.org/experiments/ENCSR000AKO/	ENCFF769AUF
K562	Rad21	https://www.encodeproject.org/experiments/ENCSR879KXD/	ENCFF057JFH
K562	Smc3	https://www.encodeproject.org/experiments/ENCSR000EGW/	ENCFF041YQC
K562	Promoter& Enhancer	https://hgdownload.soe.ucsc.edu/goldenpath/hg19/encodeDCC/wgEncodeBroadHmm/wgEncodeBroadHmmK562HMM.bed.gz	N/A
K562	RNA-seq	https://www.encodeproject.org/experiments/ENCSR000AEL/	ENCFF104VTJ

Supplementary Table 6 Summary of the twelve methods for TAD identification involved in the paper.

Method	Type	Journal	Year
Directionality Index	TAD boundary	Nature	2012
HiCSeg	TAD boundary	Bioinformatics	2014
Insulation Score	TAD boundary	Nature	2015
TopDom	TAD boundary	Nucleic Acids Research	2016
ClusterTAD	TAD boundary	BMC Bioinformatics	2017
**TADtree**	Hierarchical TADs	Bioinformatics	2016
GMAP	Hierarchical TADs	Nature Communications	2017
CaTCH	Hierarchical TADs	Genome Research	2017
**3DNetMod**	Hierarchical TADs	Nature Methods	2018
**OnTAD**	Hierarchical TADs	Genome Biology	2019
**SpectralTAD**	Hierarchical TADs	BMC Bioinformatics	2020
**TADpole**	Hierarchical TADs	Nucleic Acids Research	2020

The five methods selected for comparison are indicated in bold.

IV. Supplementary References

1 McMahan, H. B. in *Proceedings of the Fourteenth International Conference on Artificial Intelligence and Statistics.* (ed David Dunson Geoffrey Gordon, and Miroslav Dudík) 525–533 (JMLR W&CP).

2 H. Brendan McMahan, G. H., D. Sculley, Michael Young, Dietmar Ebner, Julian Grady, Lan Nie, Todd Phillips, Eugene Davydov, Daniel Golovin, Sharat Chikkerur, Dan Liu, Martin Wattenberg, Arnar Mar Hrafnkelsson, Tom Boulos, Jeremy Kubica. in *Proceedings of the 19th ACM SIGKDD international conference on Knowledge discovery and data mining.* (ed Ted E. Senator Rayid Ghani, Paul Bradley, Rajesh Parekh, Jingrui He) 1222–1230 (Association for Computing Machinery).

3 Weinreb, C. & Raphael, B. J. Identification of hierarchical chromatin domains. *Bioinformatics* **32**, 1601–1609, doi:10.1093/bioinformatics/btv485 (2016).

4 Norton, H. K. *et al.* Detecting hierarchical genome folding with network modularity. *Nat. Methods* **15**, 119–122, doi:10.1038/nmeth.4560 (2018).

5 An, L. *et al.* OnTAD: hierarchical domain structure reveals the divergence of activity among TADs and boundaries. *Genome Biol.* **20**, 282, doi:10.1186/s13059-019-1893-y (2019).

6 Cresswell, K. G., Stansfield, J. C. & Dozmorov, M. G. SpectralTAD: an R package for defining a hierarchy of Topologically Associated Domains using spectral clustering. *BMC Bioinf.* **21**, 1–19 (2020).

7 Solervila, P., Cusco, P., Farabella, I., Stefano, M. D. & Martirenom, M. A. Hierarchical chromatin organization detected by TADpole. *Nucleic Acids Res.* **48**, e39 (2020).

8 Rao, S. S. *et al.* A 3D map of the human genome at kilobase resolution reveals principles of chromatin looping. *Cell* **159**, 1665–1680, doi:10.1016/j.cell.2014.11.021 (2014).

9 Dixon, J. R. *et al.* Topological domains in mammalian genomes identified by analysis of chromatin interactions. *Nature* **485**, 376–380, doi:10.1038/nature11082 (2012).

10 Naumova, N. *et al.* Organization of the mitotic chromosome. *Science* **342**, 948–953, doi:10.1126/science.1236083 (2013).
