# Supplementary material for: TADfit is a multivariate linear regression model for profiling hierarchical chromatin domains on replicate Hi-C data
Source: Commun Biol. 2022 Jun 20;5:608. doi: 10.1038/s42003-022-03546-y (PMC9209495; doi:10.1038/s42003-022-03546-y)
Supplement: Supplementary file 3 — Description of Additional Supplementary Files [file 42003_2022_3546_MOESM3_ESM.pdf]

Description of Additional Supplementary Files

File name: Supplementary Data 1
Description: The source data behind the graphs (Fig. 2a-b, Fig. 3f-h and Fig. 5b-i) in the paper.
