# Supplementary material for: TADfit is a multivariate linear regression model for profiling hierarchical chromatin domains on replicate Hi-C data
Source: Commun Biol. 2022 Jun 20;5:608. doi: 10.1038/s42003-022-03546-y (PMC9209495; doi:10.1038/s42003-022-03546-y)
Supplement: Supplementary file 5 — Reporting Summary [file 42003_2022_3546_MOESM5_ESM.pdf]

nature portfolio

Corresponding author(s): Hongqiang Lyu

Last updated by author(s): May 9, 2022

Reporting Summary

Nature Portfolio wishes to improve the reproducibility of the work that we publish. This form provides structure for consistency and transparency in reporting. For further information on Nature Portfolio policies, see our Editorial Policies and the Editorial Policy Checklist.

Statistics

For all statistical analyses, confirm that the following items are present in the figure legend, table legend, main text, or Methods section.

n/a	Confirmed	
☒	☐	The exact sample size (*n*) for each experimental group/condition, given as a discrete number and unit of measurement
☒	☐	A statement on whether measurements were taken from distinct samples or whether the same sample was measured repeatedly
☐	☒	The statistical test(s) used AND whether they are one- or two-sided<br>*Only common tests should be described solely by name; describe more complex techniques in the Methods section.*
☐	☒	A description of all covariates tested
☐	☒	A description of any assumptions or corrections, such as tests of normality and adjustment for multiple comparisons
☐	☒	A full description of the statistical parameters including central tendency (e.g. means) or other basic estimates (e.g. regression coefficient) AND variation (e.g. standard deviation) or associated estimates of uncertainty (e.g. confidence intervals)
☐	☒	For null hypothesis testing, the test statistic (e.g. *F*, *t*, *r*) with confidence intervals, effect sizes, degrees of freedom and *P* value noted<br>*Give P values as exact values whenever suitable.*
☒	☐	For Bayesian analysis, information on the choice of priors and Markov chain Monte Carlo settings
☐	☒	For hierarchical and complex designs, identification of the appropriate level for tests and full reporting of outcomes
☒	☐	Estimates of effect sizes (e.g. Cohen's *d*, Pearson's *r*), indicating how they were calculated

Our web collection on statistics for biologists contains articles on many of the points above.

Software and code

Policy information about availability of computer code

Data collection	Both simulated and experimental Hi-C data are involved in the study. Simulated Hi-C data was generated by a modified version of the strategy given by (Mattia Forcato et al. Nature Methods, 2017) and (Aaron T.L. Lun et al. BMC Bioinformatics, 2015). Experimental Hi-C data was downloaded from Juicer data archive (James T. Robinson et al. Cell Systems, 2018). Besides, the ChIP-seq data, genomic annotation data and RNA-seq data were also downloaded from ENCODE or captured online via New WashU Epigenome Browser.
Data analysis	All the source code is freely available at https://github.com/lhqxinghun/TADfit, including our proposed R package TADfit, as well as scripts for data simulation, data normalization and data down-sampling.

For manuscripts utilizing custom algorithms or software that are central to the research but not yet described in published literature, software must be made available to editors and reviewers. We strongly encourage code deposition in a community repository (e.g. GitHub). See the Nature Portfolio guidelines for submitting code & software for further information.

Data

Policy information about availability of data

All manuscripts must include a data availability statement. This statement should provide the following information, where applicable:

- Accession codes, unique identifiers, or web links for publicly available datasets
- A description of any restrictions on data availability
- For clinical datasets or third party data, please ensure that the statement adheres to our policy

All the datasets involved in this study are presented in the manuscript or Supplementary Information, including the scripts for generation of simulated Hi-C data, the web links for the archives of experimental Hi-C data and genomic annotation data, as well as the accession codes for ChIP-seq data and RNA-seq data. These datasets are available without any restriction.

Field-specific reporting

Please select the one below that is the best fit for your research. If you are not sure, read the appropriate sections before making your selection.

☒ Life sciences ☐ Behavioural & social sciences ☐ Ecological, evolutionary & environmental sciences

For a reference copy of the document with all sections, see nature.com/documents/nr-reporting-summary-flat.pdf

Life sciences study design

All studies must disclose on these points even when the disclosure is negative.

Sample size	No sample-size calculation was performed since the experimental Hi-C data, ChIP-seq data, genomic annotation data and RNA-seq data involved in the study were downloaded from public data repositories to investigate our computational model.
Data exclusions	No data was excluded from analysis.
Replication	All attempts at replication were successful.
Randomization	Not relevant to the study since the experimental datasets involved in the study were downloaded from public data repositories.
Blinding	Not relevant to the study since the experimental datasets involved in the study were downloaded from public data repositories.

Reporting for specific materials, systems and methods

We require information from authors about some types of materials, experimental systems and methods used in many studies. Here, indicate whether each material, system or method listed is relevant to your study. If you are not sure if a list item applies to your research, read the appropriate section before selecting a response.

Materials & experimental systems

n/a	Involved in the study
☒	☐ Antibodies
☒	☐ Eukaryotic cell lines
☒	☐ Palaeontology and archaeology
☒	☐ Animals and other organisms
☒	☐ Human research participants
☒	☐ Clinical data
☒	☐ Dual use research of concern

Methods

n/a	Involved in the study
☒	☐ ChIP-seq
☒	☐ Flow cytometry
☒	☐ MRI-based neuroimaging
